# Intracisternal administration of NR2 subunit antagonists attenuates the nociceptive behavior and p-p38 MAPK expression produced by compression of the trigeminal nerve root

**DOI:** 10.1186/1744-8069-7-46

**Published:** 2011-06-08

**Authors:** Hye J Jeon, Seung R Han, Koang H Lim, Kyoung A Won, Yong C Bae, Dong K Ahn

**Affiliations:** 1 Department of Oral Physiology, School of Dentistry, Kyungpook National University, Daegu, Korea; 2 Department of Oral Anatomy, School of Dentistry, Kyungpook National University, Daegu, Korea

**Keywords:** trigeminal neuralgia, compression, trigeminal nerve root, NR2 antagonist, p38MAPK

## Abstract

**Background:**

We investigated the role of the central NMDA receptor NR2 subunits in the modulation of nociceptive behavior and p-p38 MAPK expression in a rat model with compression of the trigeminal nerve root. To address this possibility, changes in air-puff thresholds and pin-prick scores were determined following an intracisternal administration of NR2 subunit antagonists. We also examined effects of NR2 subunit antagonists on the p-p38 MAPK expression.

**Results:**

Experiments were carried out using male Sprague-Dawley rats weighing (200-230 g). Compression of the trigeminal nerve root was performed under pentobarbital sodium (40 mg/kg) anesthesia. Compression of the trigeminal nerve root produced distinct nociceptive behavior such as mechanical allodynia and hyperalgesia. Intracisternal administration of 10 or 20 μg of D-AP5 significantly increased the air-puff threshold and decreased the pin-prick scores in a dose-dependent manner. The intracisternal administration of PPPA (1, 10 μg), or PPDA (5, 10 μg) increased the air-puff threshold and decreased the pin-prick scores ipsilateral as well as contralateral to the compression of the trigeminal root. Compression of the trigeminal nerve root upregulated the expression of p-p38 MAPK in the ipsilateral medullary dorsal horn which was diminished by D-AP5, PPPA, PPDA, but not Ro25-6981.

**Conclusions:**

Our findings suggest that central NMDA receptor NR2 subunits play an important role in the central processing of trigeminal neuralgia-like nociception in rats with compression of the trigeminal nerve root. Our data further indicate that the targeted blockade of NR2 subunits is a potentially important new treatments strategy for trigeminal neuralgia-like nociception.

## Background

N-Methyl-D-aspartate (NMDA) receptors, which are among the major mediators of fast excitatory neurotransmission in the central nervous system, have an important role in long-term potentiation and depression, synaptogenesis, synaptic plasticity, and neuronal death [1,2]. The NMDA receptor (NR) family is composed of seven subunits, NR1, NR2A-D and NR3A and B, which are all products of separate genes [3]. Distinct NMDA receptor subtypes differ in their sensitivity to a variety of ligands, kinetic properties, and interactions with intracellular proteins [4]. Expression of functional recombinant NMDA receptors in mammalian cells requires the co-expression of at least one NR1 subunit, an essential channel-forming subunit, and one NR2 subunit [1,2,5]. Receptor affinity for receptor agonists and antagonists depends on the type of NR2 subunit [6,7].

Consistent with an increasing number of reports implicating the importance of the NR2 subunit in pain mechanisms, several experimental studies have demonstrated the efficacy of selective NR2 subunit antagonists [8-10]. Subcutaneous injection of formalin into the hind paw of rats, which produces typical biphasic behavioral response, shows expression of NR2 subnits including NR2A-D in the spinal cord [11]. Further, the intracisternal administration of (2R,4S)-4-(3-Phosphonopropyl)-2-piperidinecarboxylic acid (PPPA), a competitive NR2A antagonist, or (*α*R,*β*S)-α-(4-Hydroxyphenyl)-β-methyl-4-(phenylmethyl)-1-piperidinepropanol maleate (Ro25-6981), a selective NR2B antagonist, significantly suppresses the number of scratches in the second phase produced by subcutaneous injection of formalin in the vibrissa pad of rats [12]. These results suggest that NR2-containing NMDA receptors play an important role in pain transmission and that their control may provide novel therapeutic tools for future pain treatment. Although chronic pain is dependent on NMDA receptors, the clinical use of NMDA receptor antagonists is of limited application due to the side effects resulting from suppression of their physiological functions and very narrow therapeutic indices [13]. However, the spinal administration of Conantokin G, a selective inhibitor of the NR2B subunit, produces potent antinociception in formalin tests and the antinociceptive dose is approximately 20 fold lower than those required to impair motor function in a peripheral nerve injured animal model [14]. Highly potent NR2B-selective antagonists show good efficacy as pain killers and do not induce the side effects usually seen with non-selective NMDA receptor antagonists in a variety of animal models and humans [15,16]. These results suggest that selective NR2-related drugs have strong utility as analgesics without producing side effects. However, limited data are available concerning the role of central NR2 receptors in the mechanical hypersensitivity of trigeminal neuralgia.

Previous reports have demonstrated the active participation of central phospho-p38 mitogen-activated protein kinase (p-p38 MAPK) in chronic pain resulting from nerve injury. The spinal p38 MAPK, activated after spinal cord injury [17], spinal nerve ligation [18], or trigeminal nerve injury [19], has been found to contribute to development of nociceptive behavior in rats with neuropathic pain. These results postulate that central p38 MAPK pathway play an important role in the central nociceptive processing of chronic pain.

Prolonged nociceptive behavior has been introduced in rats following chronic compression of the trigeminal ganglion [20] or nerve root (unpublished data). Mechanical allodynia and hyperalgesia in the trigeminal territory of the affected nerve are also induced in this animal model, as is the upregulating of p-p38 MAPK expression in the medullary dorsal horn. The purpose of our present study was to investigate the role of the central NR2 subunits in the modulation of nociceptive behavior and expression of p38 MAPK in rats with compression of the trigeminal nerve root. In the experiments, changes in air-puff thresholds and pin-prick scores in the rats were determined following an intracisternal administration of D-2-amino-5-phosphonopentanoate (D-AP5), a non-selective NMDA site antagonist, PPPA, a competitive NR2A antagonist, Ro25-6981, a selective NR2B antagonist, or (2S,3R)-1-(Phenanthren-2carbonyl)piperazine-2,3-dicarboxylic acid (PPDA), a selective NR2C/NR2D antagonist. Changes in p-p38 MAPK expression in the medullary dorsal horn were also investigated using immunohistochemical staining and western blot analysis following the administration of NR2 subunit antagonists.

## Results

### D-AP5, a non-selective NMDA site antagonist, attenuates nociceptive behavior

**Figure 1:**
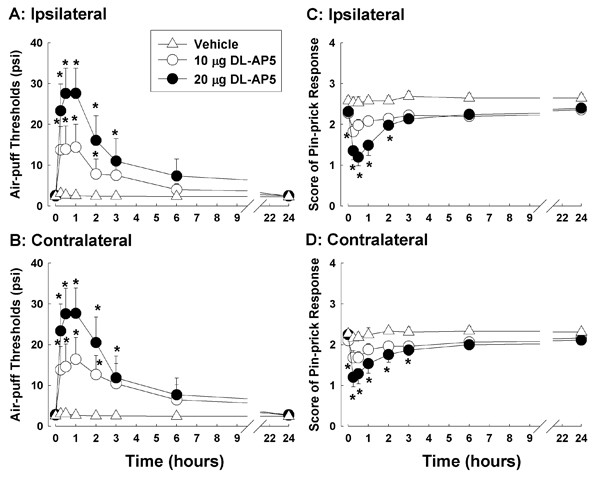
**The antinociceptive effects of D-AP5 in rats with compression of the trigeminal nerve root**. The effects of D-AP5, a non-selective NMDA site antagonist, on mechanical allodynia (A, B) and hyperalgesia (C, D) in rats with compression of the trigeminal nerve root (n = 8 in each treatment group). The intracisternal administration of vehicle did not affect air-puff thresholds in either the ipsilateral (A) or contralateral (B) side. However, 10 or 20 μg injections of AP5 increased the air-puff threshold significantly in a dose-dependent manner on both sides (A, B). These anti-allodynic effects of AP5 were maintained for three hours after injection. The intracisternal administration of 10 or 20 μg of AP5 also decreased the pin-prick scores ipsilateral (C) as well as contralateral (D) to the compression of the trigeminal nerve root. These anti-hyperalgesic effects of AP5 were completely recovered by six hours after injection. Vehicle injections did not affect pin-prick scores in the subject rats. *P < 0.05, vehicle-vs. AP-5-treated groups.

The effects of D-AP5, a non-selective NMDA site antagonist, on mechanical allodynia (A, B) and hyperalgesia (C, D) in rats with compression of the trigeminal nerve root are shown in Figure 1. The intracisternal administration of the vehicle did not affect air-puff thresholds on either the ipsilateral or contralateral side. However, 10 or 20 μg of D-AP5 increased the air-puff threshold significantly in a dose-dependent manner (F_(2, 21) _= 6.272; P < 0.01). The contralateral air-puff thresholds also increased significantly in a dose dependent manner (F_(2, 21) _= 7.355; P < 0.01) following the intracisternal administration of 10 or 20 μg of D-AP5. The anti-allodynic effects of D-AP5 were maintained for three hours after injection. The intracisternal administration of 10 or 20 μg of D-AP5 significantly decreased the pin-prick scores ipsilateral (F_(2, 21) _= 7.355; P < 0.01) as well as contralateral (F_(2, 21) _= 6.884; P < 0.01) to the compression of the trigeminal nerve root. The anti-hyperalgesic effects of D-AP5 were completely recovered from at six hours after injection. However, the vehicle injections did not affect the pin-prick scores in the rats.

### PPPA, a competitive NR2A antagonist, attenuates nociceptive behavior

**Figure 2:**
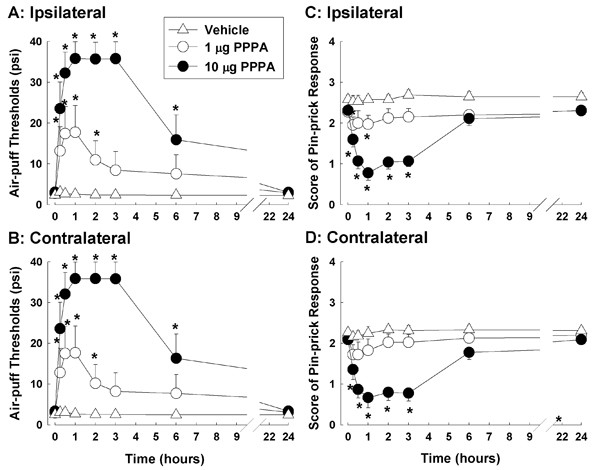
**The antinociceptive effects of PPPA in rats with compression of the trigeminal nerve root**. The effects of PPPA, a competitive NR2A antagonist, on mechanical allodynia (A, B) and hyperalgesia (C, D) in rats with compression of the trigeminal nerve root (n = 8 in each treatment group). The intracisternal administration of vehicle did not affect air-puff thresholds in either ipsilateral or contralateral side. However, 1 or 10 μg injections of PPPA significantly increased the air-puff threshold in a dose-dependent manner both ipsilateral (A) and contralateral (B) to the compression of the trigeminal nerve root. The anti-allodynic effects of PPPA were maintained for one hour after a 1 μg injection of PPPA and six hours after a 10 μg dose of PPPA. The intracisternal administration of 1 or 10 μg of PPPA also significantly decreased the pin-prick scores on both sides (C, D). The anti-hyperalgesic effects of PPPA had completely recovered by six hours after the 10 μg PPPA injection. *P < 0.05, vehicle-vs. PPPA-treated groups.

The effects of PPPA, a competitive NR2A antagonist, on mechanical allodynia (A, B) and hyperalgesia (C, D) in the rat model are shown in Figure 2. Intracisternal administration of the vehicle did not affect the air-puff thresholds on either ipsilateral or contralateral side. However, 1 or 10 μg of PPPA increased air-puff threshold significantly in a dose dependent manner both ipsilateral (F_(2, 21) _= 16.460; P < 0.001) and contralateral (F_(2, 21) _= 16.548; P < 0.001) to the compression of the trigeminal nerve root. The anti-allodynic effects of PPPA were maintained for two hours after 1 μg of PPPA and six hours after 10 μg of PPPA, respectively. The intracisternal administration of PPPA at both of these doses also significantly decreased the pin-prick scores ipsilateral (F_(2, 21) _= 18.248; P < 0.001) and contralateral (F_(2, 21) _= 13.965; P < 0.001) to the compression of the trigeminal nerve root. The anti-hyperalgesic effects of PPPA were completely recovered at six hours after the 10 μg PPPA injection. The vehicle injection did not affect the pin-prick scores.

### Ro25-6981, a selective NR2B antagonist, do not attenuate nociceptive behavior

**Figure 3:**
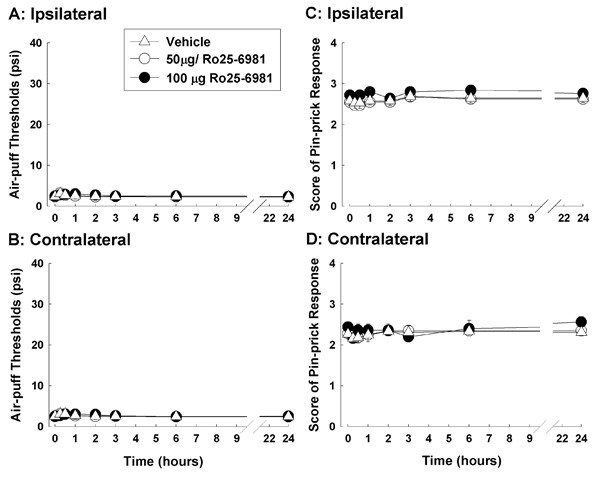
**The antinociceptive effects of Ro25-6981 in rats with compression of the trigeminal nerve root**. The effects of Ro25-6981, a selective NR2B antagonist, on mechanical allodynia (A, B) and hyperalgesia (C, D) in rats with compression of the trigeminal root (n = 8 in each treatment group). Neither the intracisternal administration of vehicle nor 50 or 100 μg of Ro25-6981 affected the air-puff thresholds on both the ipsilateral (A) and contralateral (B) sides. Moreover, injections of 50 or 100 μg of Ro25-6981 did not affect pin-prick scores on either side (C, D).

The effects of Ro25-6981, a selective NR2B antagonist, on mechanical allodynia (A, B) and hyperalgesia (C, D) in the model rats are shown in Figure 3. Neither the intracisternal administration of vehicle nor 50 or 100 μg of Ro25-6981 affected the air-puff thresholds on the ipsilateral and contralateral sides. Moreover, neither dose of Ro25-6981 affected the pin-prick scores in rats with compression of the trigeminal nerve root.

### PPDA, a selective NR2C/D antagonist, attenuates nociceptive behavior

**Figure 4:**
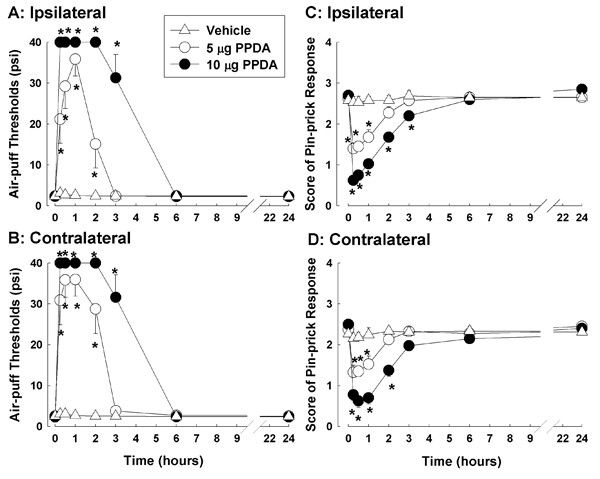
**The antinociceptive effects of PPDA in rats with compression of the trigeminal nerve root**. The effects of PPDA, a selective NR2C/NR2D antagonist, on mechanical allodynia (A, B) and hyperalgesia (C, D) in rats with compression of the trigeminal nerve root (n = 8 in each treatment group). The intracisternal administration of vehicle did not affect air-puff thresholds in either the ipsilateral (A) or contralateral (B) side. However, injections of 5 or 10 μg of PPDA produced significant anti-allodynic effects in a dose-dependent manner in both sides (A, B). The anti-allodynic effects of PPDA were maintained for two hours after injection of 5 μg of PPDA and three hours after administration of the 10 μg dose. The intracisternal administration of 5 or 10 μg of PPDA also produced significant anti-hyperalgesic effects on the ipsilateral (C) and contralateral (D) side. The anti-hyperalgesic effects of PPPA were completely recovered by six hours after injection with 10 μg of PPPA. Vehicle injections did not affect pin-prick scores in the subject rats. *P < 0.05, vehicle-vs. PPDA-treated groups.

The intracisternal injection of 5 or 10 μg of PPDA a selective NR2C/D antagonist significantly produced anti-allodynic effects in a dose dependent manner on the ipsilateral (F_(2, 21) _= 75.899; P < 0.001) and contralateral (F_(2, 21) _= 85.304; P < 0.001) sides (Figure 4A, B). These anti-allodynic effects were maintained for two hours at the 5 μg doses of PPDA and three hours at the 10 μg dose of PPDA. PPDA also produced significant anti-hyperalgesic effects on the ipsilateral (F_(2, 21) _= 26.551; P < 0.001) and contralateral (F_(2, 21) _= 16.184; P < 0.001) sides at 5 and 10 μg doses, respectively (Figure 4C, D) which were completely reversed at six hours after the 10 μg dose. The pin-prick scores were unaffected by the injection of vehicle.

### Effects of NR2 subunit antagonists on motor dysfunction

To next evaluate whether antinociceptive doses of NR2 subunit antagonists are associated with motor dysfunction, a rotarod test was performed following the intracisternal administration of AP5 (10, 20 μg), PPPA (1, 10 μg), Ro25-6981 (50, 100 μg) and PPDA (5, 10 μg) (Table 1). The intracisternal administration of vehicle did not produce any motor dysfunction. In addition, neither Ro25-6981 nor low doses of PPPA (1 μg) affected the time course of motor performance. However, other NR2 subunit antagonists produced measurable motor dysfunction. Although low doses of AP5 (10 μg) and PPDA (5 μg) caused motor dysfunction, the time course of motor performance was completely recovered to pretreatment values within 0.5 and 1 hours after injection, respectively. However, high doses of AP5 (20 μg) and PPDA (10 μg) produced significant motor dysfunction for one and three hours after injection, respectively. The time course of motor performance did not recover until six hours after the injection of 10 μg of PPPA.

**Table 1:** Effects of the intracisternal administration of NR antagonists on motor functions

Post injection time		30 min	1 hr	2 hr	3 hr	5 hr
**Chemicals**	**Dose (μg)**	**The time of motor performance (min)**				
**AP5**	**10**	60 ± 30*	**180**	**180**	**180**	**180**
	**20**	4 ± 2*	94 ± 36*	**162 ± 28**	**180**	**180**
**PPPA**	**1**	**180**	**180**	**180**	**180**	**180**
	**10**	2 ± 1*	2 ± 1*	2 ± 1*	5 ± 2*	70 ± 39*
**PPDA**	**5**	32 ± 14*	81 ± 28*	**180**	**180**	**180**
	**10**	4 ± 2*	5 ± 3*	30 ± 17*	57 ± 16*	**180**

The time course of motor performance (min) was evaluated using a rotarod test following the injection of NR2 antagonists. The time course of motor performance prior to treatment and the cut-off time is 180 min. Neither intracisternal administration of vehicle nor Ro25-6981, a selective NR2B antagonist, altered the time course of motor performance. There were 6 animals in each treatment group. * P < 0.05, compared with the vehicle-treated group.

### NR2 subunit antagonists but not Ro25-6981 reduces up-regulated p-p38 MAPK expression

**Figure 5:**
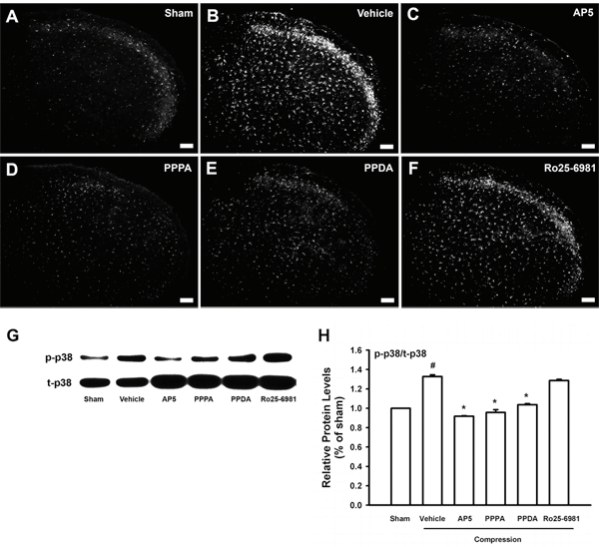
**The effects of NR2 subunit antagonists on the expression of p-p38 MAPK**. Changes in the expression of p-p38 MAPK in medullary dorsal horn of rats with compression of the trigeminal of nerve root following the administration of NR2 subunit antagonists (n = 5 in each treatment group). The intracisternal administration of vehicle did not affect p-p38 MAPK levels caused by compression of the trigeminal nerve root (B). However, the intracisternal administration of AP5 (10 μg), PPPA (1 μg), or PPDA (5 μg) decreased p-p38 MAPK expression in the medullary dorsal horn (C-E). However, Ro25-6981 (100 μg) did not affect p-p38 MAPK levels (F). Western blot analyses further confirmed that the upregulation of p-p38 MAPK was significantly suppressed by all NR2 subunit antagonists tested except for Ro25-6981 (G, H). The total p38 (t-p38) levels were used as the loading control. Scale bar, 50 μm. #P < 0.05, compared with the sham-treated group, *P < 0.05, compared with the vehicle-treated group.

The effects of NR2 subunit antagonists on the expression of p-p38 MAPK in the medullary dorsal horn of our subject rat are shown in Figure 5. Compression of the trigeminal nerve root significantly increased the expression of p-p38 MAPK immunoreactivity in the ipsilateral medullary dorsal horn. The intracisternal administration of vehicle did not affect these p-p38 MAPKs (Figure 5B), but D-AP5 (10 μg), PPPA (1 μg), and PPDA (5 μg) all suppressed the upregulation of P-p38 MAPK expression in the medullary dorsal horn (Figure 5C-E). Ro25-6981 (100 μg) had no effect (Figure 5F). Western blot analysis further demonstrated that the increased p-p38 MAPK levels was significantly reversed following the administration of all of the NR2 subunit antagonists tested except for Ro25-6981 as compared with vehicle treatment (Figure 5G, H).

## Discussion

Our present analyses first demonstrate that the intracisternal administration of NR2 subunit antagonists produces antinociceptive effects in a rat model of a compressed the trigeminal nerve root. These antinociceptive doses also suppress p38 MAPK activation in the medullary dorsal horn in subject rats. The antinociceptive potency of the NR2 subunit antagonists tested was found to be comparable, although at high doses there were motor dysfunction side effects. These results suggest that NR2 subunit antagonists have the potential utility as novel agents for trigeminal neuropathic pain relief at an appropriately managed dose.

Trigeminal neuralgia is characterized by episodes of stabbing or electrical shock-like pain. Several theories of the pathophysiology of this phenomenon have been proposed to account for its clinical features. Vascular compression of the trigeminal nerve root is known as one of the major etiological aspects of trigeminal neuralgia [21-23]. In patients with this disorder, prolonged pain can be alleviated by surgical micro-vascular decompression [21,24,25]. Although these reports have suggested that the trigeminal nerve root plays an important role in the underlying pathophysiology of trigeminal neuralgia, the etiological roles of vascular compression are inferred from clinical observations rather than from experimental manipulation. Our present study demonstrates, however, that compression of the trigeminal nerve root produces obvious nociceptive behavioral changes in rats. Compression of the trigeminal nerve root significantly decreased air-puff thresholds and increased pin-prick stimulation scores on both the ipsilateral and contralateral sides. Mechanical allodynia and hyperalgesia presented on postoperative day 3 following compression of the trigeminal nerve root and then persisted for 30 days after surgery (unpublished data).

Consistent with the present data, which demonstrated the antinociceptive effects of NR2 subunit antagonists in rats, several previous experimental studies have shown the participation of the NR2-selective NMDA receptor in pain transmission in the central nervous system [8-10]. Chronic constriction injury of the sciatic nerve in rats produces a time-dependent upregulation of NR2 subunits of the NMDA receptor within the spinal cord dorsal horn ipsilateral to the chronic constriction nerve injury [26]. NR2A or NR2B antagonists injected intracisternally have also been shown to block nociceptive scratching behavior produced by subcutaneous injection of formalin in the vibrissa pad of rats [12]. These results suggest that central NR2 subunits play an important role in pain transmission. However, our current data indicate that NR2B subunit antagonists do not attenuate nociceptive behavior, although NR2A or NR2C/D subunit antagonists do inhibit mechanical allodynia and hyperalgesia. These functional differences between NR2 subunits antagonist are supported by several previous studies. NR2A in the spinal cord has different locations at the cellular level compared with NR2B [27,28]. NR2A subunits predominate at synapses, whereas NR2B subunits are present extrasynaptically and do not participate in synaptic transmission in adult rat lamina II neurons. Neurons in lamina I have a higher mean ratio of NR2C/D subunit receptors [29] and the maximal open probability of the NMDA receptor channel also differs for each NR2 receptor subunit (NR2A-D) [30]. Moreover, functional differences between the antinociceptive actions of NR2 subunits may be caused by different animal models or species in addition to differential expression and electrical properties of NR2 subunits. Hence, differential expression or distinct pharmacological and biophysical properties of NR2 subunits may also underlie our present findings that the intracisternal administration of PPPA, PPDA, but not Ro25-6981, produces significant antinociceptive outcomes.

Previous studies have demonstrated that p38 MAPK is activated in rats with neuropathic pain caused by spinal nerve ligation [18] or spared nerve injury [31]. Moreover, pretreatment with p38 MAPK inhibitors attenuated increased p-p38 MAPK expression and nociceptive behavior. These accumulating evidences also now suggest that p38 MAPK is likely to contribute to the development of pain hypersensitivity in animal models of neuropathic pain [17-19]. Compression of the trigeminal nerve root produces significant nociceptive behavior and upregulates p-p38 MAPK expression in the ipsilateral medullary dorsal horn in the current study. These results imply that p38 MAPK is a key intracellular signal mediator involved in the development of neuropathic pain including trigeminal neuralgia-like pain caused by compression of the trigeminal nerve root. Furthermore, our present analyses show that intracisternal administration of D-AP5, PPPA, and PPDA, but not Ro25-6981 attenuates the expression of p-p38 MAPK, suggesting that specific NR2 subunit NMDA receptors participate in the central transmission of trigeminal neuropathic pain through p38 MAPK activation following compression of the trigeminal nerve root.

The cellular mechanisms of NR2 involvement in pain transmission are limited in availability. Small-diameter primary afferent fibers terminating in the dorsal horn express NMDA receptors including NR2 subunits and the activation of presynaptic NMDA receptors causes the release of substance P from primary afferents [32]. Notably, NR2 subunits are predominantly expressed on small-diameter primary afferents [33]. These results suggest that presynaptic NR2 containing NMDA receptors, which can be facilitated, prolong the transmission of nociceptive messages through the release of the neurotransmitters including substance P, calcitonin gene-related peptide, or glutamate in small-diameter primary afferent terminals [32,33]. In contrast, phosphorylated NR1 subunit is significantly upregulated in the ipsilateral dorsal horn in nerve-injured rats, whereas no differences in the expression of NR2A, NR2B, NR2C or the NR2D subunits have been reported [34]. These data suggest that the phosphorylation of the NR1 subunit correlates with the signs of neuropathy (stimulus evoked pain-like behavior) and with persistent pain following nerve injury. Hence, the role of NR1 in the central processing of trigeminal neuralgia should be studied further.

Since the spinal delivery of NMDA receptor antagonists was shown previously to inhibit the hyperexcitability of spinal cord nociceptive neurons induced by C-fiber stimulation [35,36], NMDA receptor antagonists have been shown also to effectively alleviate pain-related behavior in animal models and in clinical situations [37,38]. Although chronic pain depends on NMDA receptors, the clinical use of NMDA receptor blockers is limited by the resulting side effects [13]. In this regard, a reduced side effect profile and an improved efficacy of specific NR2-selective antagonists in animal pain models suggest that they have the potential to treat pain in human patients [39]. Encouragingly, NR2B-selective antagonists do not induce the side effects in human usually seen with non-selective NMDA receptor antagonists, even at the maximal neuroprotective doses [15,16]. Our present experiments show that low doses of NR2 subunit antagonists, which did not produce motor dysfunction or did so at levels that recovered to normal within one hour, produce a significant antinociception and decrease upregulation of p-p 38 MAPK in rats with compression of the trigeminal nerve root. However, high doses of NR2 subunit antagonists produced significant motor dysfunction upto 2 hours and 3 hours after injection. These results suggest that low concentrations of NR2 subunit antagonists may be potential treatments for trigeminal neuralgia-like nociception, although high doses of these agents should be avoided to reduce unwanted side effects in the clinic.

## Conclusions

In conclusion, we show herein that the intracisternal administration of NR2 subunit antagonists inhibits nociceptive behavioral responses and p38 MAPK activation in the medullary dorsal horn in rats with compression of the trigeminal root. These results suggest that central NR2 subunits play an important role in the central processing of trigeminal neuralgia and that a targeted blockade of the NR2 receptor is a potentially important new treatments strategy for trigeminal neuralgia.

## Methods

All procedures involving the use of animals were approved by the Institutional Care and Use Committee of the School of Dentistry, Kyungpook National University. Experiments involving the investigation of pain in conscious animals were carried out in strict accordance with the ethical guidelines of the International Association for the Study of Pain and the National Institute of Health Guide for the Care and Use of Laboratory Animals.

### Animals

Experiments were carried out on male Sprague-Dawley rats weighing between 200 and 230 g. The animals were maintained in a temperature-controlled room (23 ± 1°C) with a 12/12 hr light-dark cycle (light on at 7:00 am). All behavioral responses were measured in a blind fashion.

### Compression of the trigeminal nerve root

Compression of the trigeminal nerve root was performed under pentobarbital sodium (40 mg/kg) anesthesia. Anesthetized rats were mounted on a stereotaxic frame (Model 1404; David Kopf Instruments, Tujunga, CA) and a guide cannula (21 gauge) was implanted into the left trigeminal nerve root. The Coordinates were as follows: 7.2 mm posterior to the bregma, 2.45 mm lateral from midline, and 8.0 mm ventral from the surface of the skull. The injector (24 gauge) was connected to a 100 μl Hamilton syringe with polyethylene tube (PE 50, Clay Adams, Parsippany, NJ) and was preheated in the warm water bath (38°C). A 4% agar solution (10 μl) was then administrated for 5 sec through the injector, which extended 1 mm beyond the end of a guide cannula. The injected agar was located on the dorsal surface of the trigeminal nerve root to induce compression without any injury during operation. The sham group received no agar injections. After the injector and guide cannula were removed, the incision was sutured. At the end of each experiment, the compressed site of trigeminal nerve root was confirmed. The animals were deeply anesthetized with a lethal dose of pentobarbital sodium and were perfused through the heart with phosphate buffered saline. After perfusion, the brain was removed and injection sites were examined using a stereoscope. Only data from rats showing clear compression sites on the trigeminal nerve root were used in the final analysis.

### Evaluation of facial nociceptive behavior following compression of the trigeminal nerve root

#### Mechanical allodynia

For behavioral observations, each rat was placed in a customized observation cage in a darkened and noise-free room and acclimated for at least 30 min. An evaluation of withdrawal behavioral responses was performed after the application of 10 successive trials of constant air-puff pressure (4 sec duration and 10 sec intervals) on freely moving rats, as previously described [20,40]. The intensity and intervals of the air-puffs were controlled with a pneumatic pump module (BH2 system; Harvard Apparatus, Holliston, MA). The air-puffs were applied through a 26 gauge metal tube (length, 10 cm) located 1 cm from the skin and the cut-off pressure was 40 psi. Allodynia was defined as the decrease in the intensity of the air-puff threshold when rats attempted to escape or showed an aggressive behavioral response to air-puff stimulation. The thresholds were determined as the air-puff pressure at which each rat responded in 50% of the trials. Naïve animals did not respond to less than 40 psi.

#### Mechanical hyperalgesia

Mechanical hyperalgesia was determined using the pinprick test after surgery as previously described [20,40]. Briefly, the rats were acclimated in an observation cage before testing. The tip of a 0.2 mm diameter blunted acupuncture needle (Needle No.3, Serin, Tokyo, Japan) was pushed against the vibrissa pad, until the needle was slightly bent (the skin was dimpled but not penetrated). We estimated the rat responses to five pin-prick stimulus which were scored on an ordinal scale [20,40,41] as follows: no response = 0; detection = 1; detection and withdrawal = 2; detection, withdrawal, and escape or attacking movements = 3; and all of the characteristics of response 3, but with prolonged ipsilateral facial grooming (> 3 strokes) = 4.

### Effects of NR2 subunit antagonists on compression of the trigeminal nerve root-induced mechanical allodynia and hyperalgesia

The effects of NR2 subunit antagonists on mechanical allodynia and hyperalgesia were evaluated on postoperative day 14. Animals that displayed mechanical allodynia and hyperalgesia following compression of the trigeminal nerve root were used for assessments. For intracisternal injection, individual rats were anesthetized with mixture of ketamine (0.2 g/kg) and xylazine (0.02 g/kg). Anesthetized rats were individually mounted on a stereotaxic frame and a polyethylene tube (PE10, CalyAdams, Parsippany, NJ) was implanted for intracisternal injection on postoperative day 11, as described previously [42-45]. The polyethylene tube was inserted through a tiny hole made in the atlantooccipital membrane and dura, using a 27 gauge syringe needle. The tip of the cannula was placed at the obex level. The polyethylene tube was subcutaneously led to the top of the skull and secured in place by a stainless steel screw and dental acrylic resin. In the 72 hour recovery period after surgery, NR2 subunit antagonists were intracisternally administered. For confirmation of the placement of the intracisternal cannula and the extent of the spread of the drugs, pontamine sky blue dye was injected at the end of the tests. The animals that showed motor dysfunction or mal-position of the catheter after intracisternal catheterization were excluded from the analysis. Mechanical allodynia and hyperalgesia was determined 15, 30, 60, 120, 180, 360 and 1440 min after intracisternal administration of NR2 subunit antagonists.

### Administration of chemicals

The following compounds were used for intracisternal injection of D-2-amino-5-phosphonopentanoate (D-AP5), a non-selective NMDA site antagonist, (2R,4S)-4-(3-Phosphonopropyl)-2-piperidinecarboxylic acid (PPPA), a competitive NR2A antagonist, (*α*R,*β*S)-α-(4-Hydroxyphenyl)-β-methyl-4-(phenylmethyl)-1-piperidinepropanol maleate (Ro25-6981), a selective NR2B antagonist, or (2S,3R)-1-(Phenanthren-2carbonyl)piperazine-2,3-dicarboxylic acid (PPDA), a selective NR2C/NR2D antagonist. All NMDA receptor related chemicals were purchased from Tocris (Bristol, U.K.) and dissolved in normal saline.

### Immunohistochemistry

Some rats (n = 5 per group) were perfused through the ascending aorta with 0.9% saline, followed by 4% paraformaldehyde in 0.1 M PB (pH 7.4) under anesthesia 3 hours after administration of NR2 subunit antagonists or vehicle. The caudal medulla were then dissected out, postfixed in the same fixative at 4°C overnight, and replaced with 30% sucrose in 0.1 M PB overnight. Transverse frozen sections (free-floating, 30 μm) were cut in a cryostat and processed for immunofluorescence. All sections were blocked with 5% goat serum in 0.2% Triton X-100 for 1 hour at room temperature and then incubated overnight at 4°C with a rabbit polyclonal phospho p38 antibody (1:300, Cell Signaling Technology, Danvers, MA). The sections were then incubated with Alexa 555-conjugated rabbit IgG antibody (1:400, Invitrogen, Carlsbad, CA) for 2 h at room temperature. The stained sections were then observed under fluorescence microscope (Zeiss Axioplan, Carl Zeiss, Germany).

### Western blotting

Some rats (n = 5 per group) were sacrificed under anesthesia 3 hours after administration of NR2 subunit antagonists or vehicle. The dorsal part of caudal medulla was then rapidly removed and frozen in liquid nitrogen. Samples were sonicated with Biorupture (Cosmo Bio., Tokyo, Japan) in lysis buffer containing protease and phosphatase inhibitor cocktail (Thermo Scientific, Rockford, IL). Protein concentrations of samples were measured using a fluorometer (Invitrogen). For western blotting, total proteins (30 μg) were separated by 4-12% gradient NuPAGE Novex Bis-Tris gel (Invitrogen) and transferred onto PVDF membrane by using the iBlot Dry blotting system (Invitrogen). The membranes were then blocked with 5% non-fat milk in TBS with 0.1% Tween 20 for 1 hour at room temperature and then incubated with p-p38 or total p38 (both diluted 1:1000; Cell Signaling Technology) as loading control at 4°C overnight. The blots were then incubated with goat anti-rabbit horseradish peroxidase for 1 hour at room temperature. Membranes were developed using the SuperSignal West Femto substrate (Pierce, Rockford, IL), and exposed to X-ray film. A computer image analysis system (ImageJ; NIH, Bethesda, MD) was used for quantification of the specific bands.

### Rotarod test

Changes in motor performance, after the intracisternal administration of D-AP5, PPPA, Ro 25-6981, or PPDA, were measured using a rotarod (Ugo Basile, Comerio-Varese, Italy), as described previously [20,40,46,47]. The rotarod speed was increased to 16 rpm with the maximum time spent on the rod set at 180 seconds. Rats received two or three training trials on two separate days prior to testing for acclimatization. On the experimental days, the time course of the motor performance was examined before and after the intracisternal administration of NMDA receptor antagonists.

### Statistics

Differences between groups were compared using analysis of repeated measures ANOVA followed using LSD post hoc analysis. Comparisons of p-p38 MAPK expression between NR2 subunits antagonists were performed using one-way ANOVA followed using LSD post hoc analysis. In all statistical comparisons, a P value of < 0.05 was used as the criterion for statistical significance. All data are presented as the mean ± SEM.

## Abbreviations

D-AP5: D-2-amino-5-phosphonopentanoate; NMDA: N-Methyl-D-aspartate; p-p38 MAPK: phospho-p38 mitogen-activated protein kinase; PPDA: (2S,3R)-1-(Phenanthren-2carbonyl)piperazine-2,3-dicarboxylic acid; PPPA: (2R,4S)-4-(3-Phosphonopropyl)-2-piperidinecarboxylic acid; Ro25-6981: (*α*R,*β*S)-α-(4-Hydroxyphenyl)-β-methyl-4-(phenylmethyl)-1-piperidinepropanol maleate.

## Competing interests

The authors declare that they have no competing interests.

## Authors' contributions

HJJ and SRH carried out the experiment and drafted the manuscript. KHL and KAW participated in the design of the study. YCB help conceive of the study, and participated in its design. DKA coordinated and supervised the experiments, analyzed the data and wrote the manuscript. All authors read and approved the final manuscript.
